# microRNA profile datasets of murine macrophages infected with different strains of Leptospira spp

**DOI:** 10.1038/sdata.2018.171

**Published:** 2018-08-21

**Authors:** Leandro E. Garcia, Erivelto C. A. Junior, Jaqueline P. Bragato, Larissa M. Melo, Valéria F. M. Lima, Juliana R. Peiró, Daniel R. Arnold, Márcia Marinho, Flavia L. Lopes

**Affiliations:** 1 Department of Support, Production and Animal Health, São Paulo State University (Unesp), School of Veterinary Medicine, Araçatuba, SP 16050-680, Brazil; 2 Department of Clinics, Surgery and Animal Reproduction, São Paulo State University (Unesp), School of Veterinary Medicine, Araçatuba, SP 16050-680, Brazil

**Keywords:** Infection, Gene silencing

## Abstract

MicroRNAs play an important role in the regulation of immune responses. The influence of epigenetic mechanisms, particularly RNA-mediated post-transcriptional regulation of host immune responses has been proven vital following infections by different pathogens, and bacteria can modulated host miRNAs. Global miRNA expression analysis from macrophages infected *in vitro* with different strains of *Leptospira* spp was performed using miRNA 4.1 microarray strips. Leptospirosis is a bacterial zoonosis of global importance, responsible for significant morbidity and mortality worldwide. Despite considerable advances, much is yet to be discovered about disease pathogenicity, particularly in regards to host-pathogen interactions. We present here a high-quality dataset examining the microtranscriptome of murine macrophages J774A.1 following 8h of infection with virulent, attenuated and saprophyte strains of *Leptospira*. Metadata files were submitted to the Gene Expression Omnibus (GEO) repository.

## Background & Summary

It is known that during bacterial infection, miRNAs play an important role in the regulation of host immune response^1,2^. These small molecules of 21 nucleotides have a vital function in the post-transcriptional regulation of gene expression. Through base pairing, miRNAs bind to complementary sequences of their target mRNAs leading to degradation or translational repression^3–5^. Each miRNA can control hundreds of target genes^6^. A range of pathogens (viruses, parasites and bacteria) can affect expression of host miRNA^1,2,7^. For this reason, pathogen-induced gene modulation of host cells is essential to understanding the pathophysiology of diseases. In this study, we evaluate, for the first time, global miRNA expression in macrophages infected with different strains of *Leptospira* spp.

*Leptospira interrogans* is a highly invasive gram-negative spirochete that leads to development of leptospirosis. These bacteria can occur in urban and rural environments, surviving mainly in water. Currently, the rate of mortality can reach 60.000 deaths per year^8,9^. In the literature 12 species and more than 250 serotypes of *Leptospira* are described, varying in pathogenicity^10,11^. During early infection, antibiotics are effective, however most vaccines available for veterinary use provide limited protection against more than 250 pathogenic *Leptospira* serovars^12,13^.

**Figure 1:**
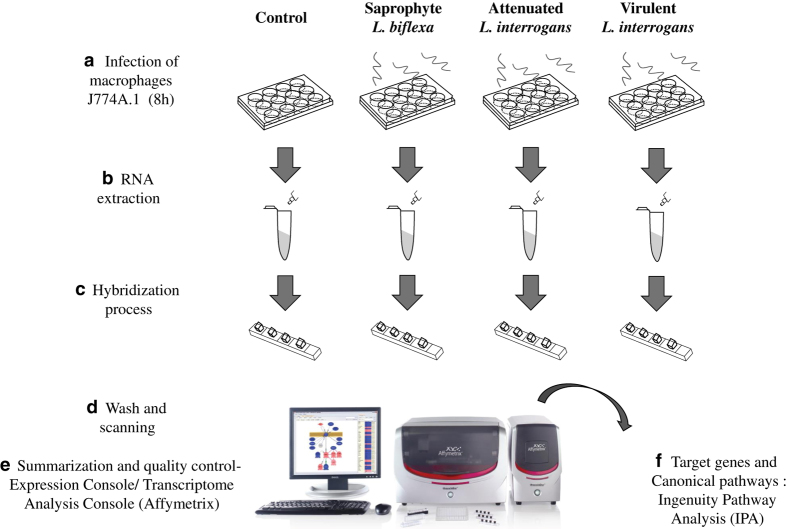
Illustration of experimental design. Cell lineage J774A.1 of murine macrophages was cultured to a confluent monolayer. Infection was performed adding 100:1 bacteria:cell to the macrophages. (**a**) Treatments, analyzed in triplicate, were carried as follows: infection of macrophages with a virulent strain of *L. interrogans*, with an attenuated strain of *L. interrogans*, and with an saprophyte strain *L. biflexa* and non-infected macrophages as controls. All treatments were incubated in fresh RPMI medium, without antibiotics, for 8 h at 37 °C, 5% C02. (**b**) Following this period, total RNA was immediately extracted, (**c**,**d**) hybridization of samples to the strips was carried at 48 °C for 20 h, strips were then washed, stained and scanned using the GeneAtlas® System (Affymetrix). (**e**) Raw intensity values were background corrected, log2 transformed and then quantile normalized by the software Expression Console (Affymetrix) using the Robust Multi-array Average (RMA) algorithm. Statistical analysis was performed in the TAC software (Affymetrix) and cel files were submitted to Gene Expression Omnibus repository (GEO). (**f**) Target genes and Pathway analysis was performed in the Ingenuity Pathway Analysis (Qiagen).

Macrophages are responsible for bacterial phagocytosis in mammals and are important cells in leptospiral infection^14,15^. Host/pathogen interaction can modify gene expression profiles in the host with leptospirosis^16^. Our goal was to contribute to the understanding of pathogen-mediated control of host gene expression by identifying miRNAs modulated by saprophyte, attenuated or virulent strains of *Leptospira* in macrophages compared to non-infected control cells. Through the use of microarray technology, we generated microtranscriptome datasets following 8 hours of infection. We worked with the hypothesis that *Leptospira* infection modulates macrophageal expression of miRNAS, and that bacterial virulence affects this modulation. Our study suggests that post-transcriptional regulation by miRNAs plays a role in host response to infection in leptospirosis. Here, we describe detailed information on the experimental design (Fig. 1) and generation of our datasets (Data Citation 1). This data descriptor is an extend version of the methodology described in a related paper^17^, with the objective of disseminating the raw data produced in this experiment. These raw data can be a valuable resource for further bioinformatics investigation of biological pathways associated with pathogenicity, leading to the identification of novel targets for therapy.

## Methods

### Cell culture

Murine macrophage cell line J774A.1 was provided by the Paul Ehrlich cell bank, Rio de Janeiro, Brazil. This cell lineage was maintained in RPMI-1640 media (Sigma, USA), supplemented with 10% heat-inactivated fetal bovine serum (Gibco, USA), 100 ug/mL streptomycin (Sigma, USA), 0.03% L-glutamine solution (Sigma, USA) and 100UI/mL of penicillin. Cells were incubated at 37 °C, 5% CO_2_ until formation of a confluent monolayer in 6-well cell culture plates (3 cm/well).

### Bacterial culture

All strains of *Leptospira* used in this study, *Leptospira interrogans* serovar Copenhageni (FIOCRUZ L1-130) as a virulent strain, *L. interrogans* serovar Copenhageni M20 as an attenuated strain and *Leptospira biflexa* serovar Patoc (FIOCRUZ -Patoc I) as a saprophyte strain, were kindly provided by the Laboratory of Preventive Veterinary Medicine of University of São Paulo (USP). Attenuation of M20 strain was done by successive passages (>200), according with reference^18–20^. All strains were maintained in Fletcher’s semi solid medium, and incubated at 30 °C. Virulence of *L. interrogans* L1-130 was preserved by intraperitoneal inoculation in hamsters (*Mesocricetus auratus*) with kidney recovery, following previously published guidelines^19^. Before infection, all strains were counted in a Petroff–Hausser counting chamber (Fisher Scientific)^21^. Project had the approval of the Ethics Committee for Animal Use (FOA-FMVA Unesp), under protocol number 2015-00895.

### Infection of Macrophages

After the formation of monolayers with >90% confluency, cells were washed with sterile phosphate buffer solution (pH 7,2) for removal of antibiotics and non-adherent cells. *L. interrogans* and *L. biflexa* were centrifuged, for removal of their growth media, and resuspended in RPMI-1640 medium (Sigma), and added to macrophages (100:1 bacteria:cell). Experimental groups were devised as follows: infection of macrophages with a virulent strain (*L. Interrogans*; n=3), infection with attenuated strain (*L. interrogans*; n=3), infection with saprophyte strain (*L. biflexa*; n=3) and non-infected macrophages (control; n=3). All treatments were incubated in fresh RPMI medium, without antibiotics, for 8h at 37 °C, 5% CO_2_. Following this period, RNA extraction was immediately performed.

### miRNA extraction and Quantification

Extraction of total RNA from macrophages was conducted using a miRVana miRNA Isolation Kit (Ambion, Austin, TX, USA) following the manufacturer’s instructions. RNA samples were immediately stored at −80 °C. Quantification was performed using a spectrophotometer (Nanodrop ND-20, Thermo Scientific, Wilmington, DE, USA) and quality of samples was assessed with capillary electrophoresis (Bioanalyzer 2100 Agilent, Santa Clara, CA, USA).

### Microtranscriptome Array

**Figure 2:**
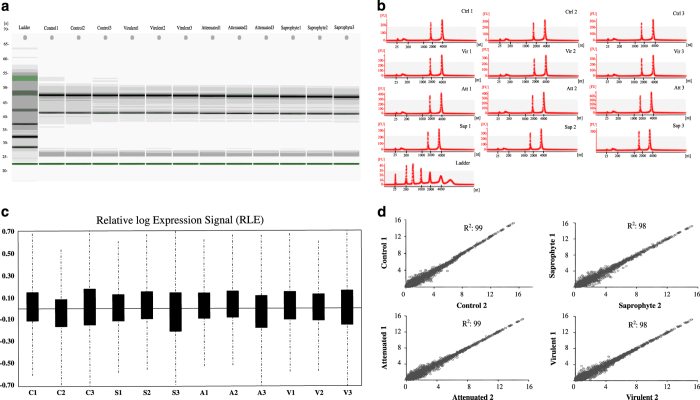
RNA and microarray quality control and summarization. (**a**,**b**) Bioanalyzer gel image and graphic of all RNA samples; Control (Ctrl); Virulent (Vir); Attenuated (Att) and Saprophyte (Sap), used for microarray analysis. The 28S and 18s distinctive ribosomal RNA bands are observed for all samples. (**c**) Values of relative log expression signal RMA-DABG between treatments. (**d**) Signal intensity correlation analysis within groups.

**Figure 3:**
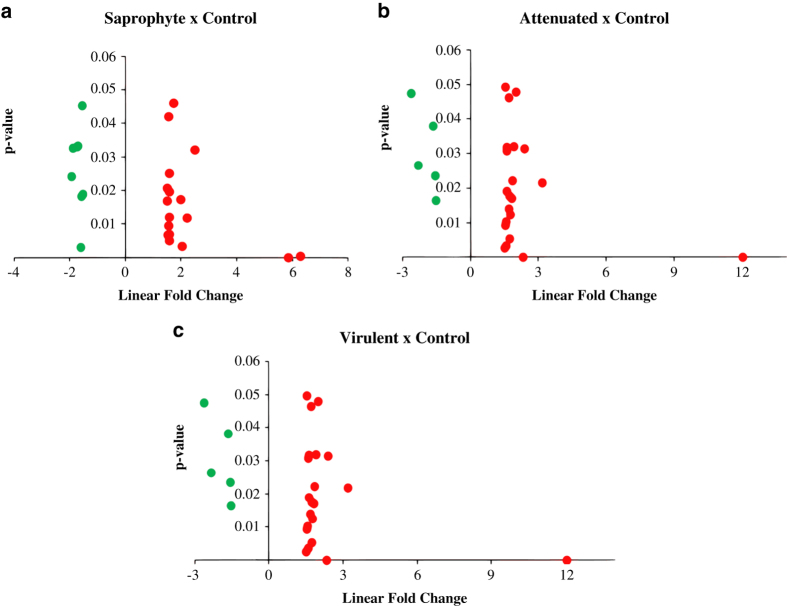
Distribution of differentially expressed miRNAs. (**a**-**c**) Upregulated (red) and downregulated (green) miRNAs in macrophages infected with virulent strains compared to control non-infected, plotted by p-value <0.05 and linear fold change. (n=3/treatment).

FlashTag Biotin HSR RNA Labeling Kit and the Affymetrix miRNA 4.1 Array strip (Affymetrix, Santa Clara, California, EUA) were used to analyze the expression of 3195 murine specific probes. ELOSA (enzyme-linked oligosorbent assay) quality control assay was run for all samples, and hybridization to the strips was carried at 48 °C for 20 h. Strips were processed and scanned in the GeneAtlas System (Affymetrix). Raw intensity values (cel files) were background corrected, log2 transformed and then quantile normalized by the software Expression Console (Affymetrix) using the Robust Multi-array Average (RMA) algorithm. Figure 2c shows the relative signal of probes. Also in expression console software, we performed a correlation with linearized signal intensity values for the samples within treatment groups, showing a strong correlation coefficient between samples (Fig. 2d). Figure 3 shows plots of p-values for miRNAs (p-value<0.05) up- and downregulated by the different leptospires in comparison to non-infected control samples.

## Data Records

The cel and arr (strip information) files produced by microarray were deposited at the Gene Expression Omnibus repository (Data Citation 1). Through the Expression Console (Affymetrix), a free software, the cel files were normalized and chp file were generated for the identification of differentially expressed miRNAs. Samples used in the study are shown in Table 1.

## Technical Validation

### RNA quality control

Quality control of RNA samples was performed prior to microarray experiment using capillary electrophoresis (Bioanalyzer 2100 Agilent, Santa Clara, CA, USA), using the Eukaryote Total RNA Nano kit. All samples used for microarray analysis had a RNA integrity number (RIN) of 10 (Fig. 2a,b).

### Microarray summarization and quality control

The Gene Atlas equipment scans hybridized strips and converts the readings into raw intensity values (cel files). These files were background corrected, log2 transformed and then quantile normalized by the software Expression Console (Affymetrix) using the Robust Multi-array Average (RMA) algorithm. Figure 2c shows the relative signal of probes. Also in the expression console software, we performed a correlation with linearized signal intensity values for the samples within treatment groups, showing a strong correlation coefficient (R^2^) between samples (Fig. 2d). Figure 3 shows plots of p-values for miRNAs up and downregulated (p-value<0.05) by the different strains in comparison to non-infected control samples.

### Validation of microarray results by qRT-PCR

**Figure 4:**
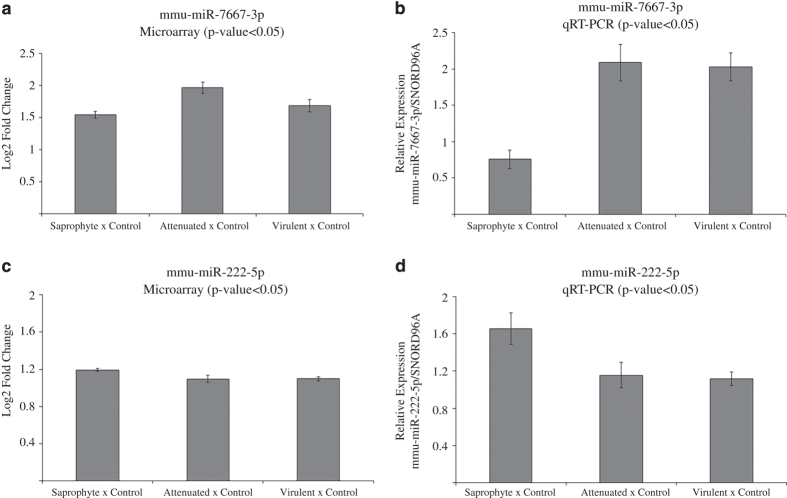
Microarray and qPCR fold change values of validated miRNAs. (**a**) mmu-miR-7667-3p microarray fold change. (**b**) mmu-miR-7667-3p qPCR fold change. (**c**) mmu-miR-222-5p microarray fold change. (**d**) mmu-miR-222-5p qPCR fold change. Quantification based on a standard curve and adjusted to SNORD96A (housekeeping). Statistical analysis between two groups (infected vs. control) was performed with ANOVA (p-value <0.0001) followed by Tukey´s Range Test.

Validation of miRNAs in infected macrophages (saprophyte, attenuated and virulent strains) and non-infected controls was done using the miScript miRNA PCR System (Qiagen-Valencia, CA, USA) for preparation of cDNA and realtime PCR, according to the manufacturer’s instructions. Validated primers were purchased from Qiagen. PCR was performed using a Stratagene QPCR Systems Mx3005P (Agilent Technologies, Santa Clara, CA, USA) Expression levels were determined using standard curves at each individual run, and the expression of candidate miRNAs is presented as a ratio to the control miRNA SNORD96A. Real time PCR data were analyzed using least-squares analysis of variance and the general linear model procedures of SAS (SAS Institute, Cary, NC, USA; p<0.01). Comparison of means was done using Tukey's range test, and significance was set at p<0.05, (Fig. 4a-d).

## Usage Notes

Raw data (cel files) can be normalized by Expression Console and Transcriptome Analysis Console (TAC), both are free softwares from Affymetrix. They perform statistical testing and present results as fold change for differentially expressed miRNAs. For miRNAs, TAC software can also identify target genes reported by Affymetrix (NetAffx).

## Additional information

**How to cite this article**: Garcia, L. E. *et al*, microRNA profile datasets of murine macrophages infected with different strains of Leptospira spp. *Sci. Data* 5:180171 doi: 10.1038/sdata.2018.171 (2018).

**Publisher’s note**: Springer Nature remains neutral with regard to jurisdictional claims in published maps and institutional affiliations.

## Supplementary Material



## Figures and Tables

**Table 1:** Dataset and sample description across treatment groups.

**GSM-ID**	**Sample Name**	**Oganism**	**Treatment description**	**Molecule**	**Technology**
GSM2817991	Control 1	Mus musculus	Macrophages_non-infected_8h_rep1	miRNA	Microarray
GSM2817992	Control 2	Mus musculus	Macrophages_non-infected_8h_rep2	miRNA	Microarray
GSM2817993	Control 3	Mus musculus	Macrophages_non-infected_8h_rep3	miRNA	Microarray
GSM2817994	Saprophyte 1	Mus musculus	Macrophages infected with saprophyte strain_8h_rep1	miRNA	Microarray
GSM2817995	Saprophyte 2	Mus musculus	Macrophages infected with saprophyte strain_8h_rep2	miRNA	Microarray
GSM2817996	Saprophyte 3	Mus musculus	Macrophages infected with saprophyte strain_8h_rep3	miRNA	Microarray
GSM2817997	Attenuated 1	Mus musculus	Macrophages infected with attenuated strain_8h_rep1	miRNA	Microarray
GSM2817998	Attenuated 2	Mus musculus	Macrophages infected with attenuated strain_8h_rep2	miRNA	Microarray
GSM2817999	Attenuated 3	Mus musculus	Macrophages infected with attenuated strain_8h_rep3	miRNA	Microarray
GSM2818000	Virulent 1	Mus musculus	Macrophages infected with virulent strain_8h_rep1	miRNA	Microarray
GSM2818001	Virulent 2	Mus musculus	Macrophages infected with virulent strain_8h_rep2	miRNA	Microarray
GSM2818002	Virulent 3	Mus musculus	Macrophages infected with virulent strain_8h_rep3	miRNA	Microarray
